# *Chlamydia trachomatis* TmeB antagonizes actin polymerization via direct interference with Arp2/3 activity

**DOI:** 10.3389/fcimb.2023.1232391

**Published:** 2023-07-07

**Authors:** Kaylyn R. Scanlon, Gabrielle Keb, Katerina Wolf, Travis J. Jewett, Kenneth A. Fields

**Affiliations:** ^1^ Division of Immunity and Pathogenesis, Burnett School of Biomedical Sciences, College of Medicine, University of Central Florida, Orlando, FL, United States; ^2^ Department of Microbiology, Immunology and Molecular Genetics, University of Kentucky College of Medicine, Lexington, KY, United States

**Keywords:** type III secretion, cytoskeleton, invasion, actin, pathogenesis

## Abstract

*Chlamydia trachomatis* is an obligate intracellular pathogen that actively promotes invasion of epithelial cells. A virulence-associated type III secretion system contributes to chlamydial entry and at least four effectors have been described that are deployed during this time. Two of these invasion-related effectors, the translocated membrane-associated effectors A and B (TmeA and TmeB), are encoded in a bi-cistronic operon. TmeA directly activates host N-WASP to stimulate Arp2/3-dependent actin polymerization. According to current working models, TmeA-mediated N-WASP activation contributes to invasion. TmeB has not been functionally characterized. Unlike a *tmeA* null strain, loss of *tmeB* does not impact invasion efficiency of *C. trachomatis*. Using strains deficient for multiple genes, we provide evidence that TmeA is dispensable for invasion in the absence of TmeB. Our data indicate that overabundance of TmeB interferes with invasion and that this activity requires active Arp2/3 complex. We further show that TmeB is capable of interfering with Arp2/3-mediated actin polymerization. In aggregate, these data point to opposing functions for TmeA and TmeB that manifest during the invasion process. These studies raise intriguing questions regarding the dynamic interplay between TmeA, TmeB, and branched actin polymerization during chlamydial entry.

## Introduction

*Chlamydia trachomatis* is a prevalent obligate intracellular bacterial pathogen that is responsible for genital and ocular diseases. Genital-specific serotypes (Serovars D-K) collectively are the most common bacterial sexually transmitted disease world-wide (Kreisel et al., 2021). Untreated *C. trachomatis* serovar D-K infections may lead to inflammation of the genitourinary tract tissues resulting in urethritis, proctitis, cervicitis salpingitis, pelvic inflammatory disease (PID) and perihepatitis (Gottlieb et al., 2010). *C. trachomatis* serovars L1-3 also infect genital tracts, but infections manifest as the more invasive disease manifestation lymphogranuloma venereum (LGV). *C. trachomatis* (serovars A-C) is also the leading cause of preventable blindness. Additional species pathogenic for humans include the respiratory pathogen *C. pneumoniae* and the zoonotic avian pathogen C. *psittaci* (Schachter, 1999). All *Chlamydia* spp exhibit a unique biphasic developmental cycle (AbdelRahman and Belland, 2005) that alternates between the extracellular-adapted, non-replicating yet infectious, elementary body (EB) and the vegetative, yet non-infectious, reticulate body (RB).

During infection, chlamydial EBs engage new host cells via initial electrostatic interactions followed by a more intimate and irreversible association (Carabeo and Hackstadt, 2001). Attachment is thought to proceed via a collection of host cell receptors that includes epithelial growth factor receptor (EGFR), fibroblast growth factor receptor (FGFR), mannose receptor (MR), protein disulfide isomerase (PDI), and mannose-6 phosphate (M6P) receptor (Elwell et al., 2016). Following receptor engagement, chlamydiae can invade host cells using multiple, likely redundant, pathways. A consensus model has emerged where entry pathways include pathogen-initiated endocytosis (Caven and Carabeo, 2019), receptor-mediated endocytosis (Gitsels et al., 2019), and capture by preformed filopodia followed by macropinocytosis (Ford et al., 2018; Jarsch et al., 2020). Efficient chlamydial entry requires a type III section system (T3SS). Anti-host, substrate proteins are pre-packaged in EBs, secreted during attachment and entry, and referred to as “early”- or “invasion-related” effectors (Mueller et al., 2014). Following internalization, the EB resides in a specialized vacuole called an inclusion and differentiates into the replicative competent RB. Development is concluded after asynchronous conversion of RBs back to EBs and subsequent exit from host cells *via* lysis or extrusion (Hybiske and Stephens, 2007).

Four chlamydial early effectors share the secretion chaperone Slc1 (Brinkworth et al., 2011; Pais et al., 2013) that likely governs the order of their secretion (Chen et al., 2014). These include the translocated actin recruiting phosphoprotein (Tarp), translocated early phosphor-protein (Tepp), and translocated membrane-associated effectors A and B (TmeA, TmeB) (Elwell et al., 2016), all of which are translocated into the cell within minutes of attachment (Clifton et al., 2004; Hower et al., 2009; Chen et al., 2014; Mueller and Fields, 2015). Both translocated Tarp (Jewett et al., 2008; Mehlitz et al., 2008) and Tepp (Chen et al., 2014) are phosphorylated by host cell kinases. Tarp directly nucleates actin monomers, polymerizes actin, bundles actin filaments (Jewett et al., 2006; Jiwani et al., 2013), and cooperates with branched actin polymerization (Rotty et al., 2013) mediated by the actin related protein complex (Arp2/3) (Jiwani et al., 2012). Tepp recruits phosphoinositide 3-kinases (PI3K) (Carpenter et al., 2017) and the CRK-Like proto-oncogene adapter protein (CrkL) (Chen et al., 2014) to direct host cell signaling to promote chlamydial development. TmeA associates with host membranes through a discreet membrane localization domain (Bullock et al., 2012) while the C-terminus interacts with the host scaffolding protein AHNAK (Hower et al., 2009). TmeA also directly activates host Neural-Wiskott Aldrich Syndrome Protein (N-WASP) to increase Arp2/3-directed actin polymerization (Keb et al., 2021). Residues 118-126 are required for TmeA to bind the GBD domain of N-WASP (Faris et al., 2020). TmeA therefore bypasses the need for N-WASP activation by the RhoGTPase Cdc42. N-WASP activation involves unfolding the protein, and freeing the C-terminal, Arp2/3-activating VCA domain. This domain consists of a verprolin homology sequence (V), a central (C) sequence and an acidic (A) sequence (Kelly et al., 2006). The VC domain is capable of binding monomeric actin, while the CA domain binds to and activates Arp2/3 (Padrick et al., 2011). Analysis of effector-deficient strains indicate that both Tarp (Ghosh et al., 2020) and TmeA (McKuen et al., 2017) are required for efficient *C. trachomatis* invasion and function synergistically (Faris et al., 2020; Keb et al., 2021) to accomplish entry.

Loss of *tmeB* does not impact invasion or intracellular growth (McKuen et al., 2017) and the function of TmeB has yet to be elucidated. The TmeB encoding gene is co-expressed with *tmeA* from a bi-cistronic operon and is also capable of associating with host cell membranes (Mueller and Fields, 2015). Given the operon structure, we hypothesized that TmeB may be functionally related to TmeA. We therefore sought to determine whether TmeB, similar to TmeA, played a role in actin dynamics during entry. In this report, we examined EB invasion kinetics of *C. trachomatis* strains lacking TmeB or expressing high levels of TmeB. We show that high levels of TmeB correlate with decreased rates of chlamydial entry. Moreover, we demonstrated that TmeB inhibits the activation of the host Arp2/3 complex in *in vitro* actin polymerization assays. Collectively, we provide evidence that TmeB functions during *Chlamydia* invasion by negatively regulating Arp2/3-mediated actin polymerization.

## Methods

### Cell culture and organisms

*C. trachomatis* serovar L2 (434/Bu) was used as the parent strain in these studies. Previously described strains include *C. trachomatis* Δ*tarp* (Ghosh et al., 2020), Δ*tmeB* (Mueller et al., 2016), Δ*tepp* (Keb et al., 2021), Δ*tmeA* (Mueller et al., 2016) (referred to herein as Δ*tmeA* polar), and *tmeA-lx* (McKuen et al., 2017) (referred to herein as non-polar Δ*tmeA*). *C. trachomatis ΔtmeAB* and Δ*tmeAB-tarp* were generated via FRAEM (Mueller et al., 2016) and are described below. Mobilization of respective plasmids into *C. trachomatis* L2 was achieved using CaCl_2_-mediated chemical transformation (Wang et al., 2012). Manipulations leveraging fluorescence reporting to generate engineered strains were accomplished according to established protocols (Mueller et al., 2017; Wolf et al., 2019). Chlamydiae were routinely maintained in either HeLa 229 epithelial cell monolayers (CCL-1.2; ATCC) or McCoy cell monolayers (CRL-1696; ATCC). Unless otherwise indicated, all cultures were grown in RPMI 1640 medium containing 2 mM L-glutamine (Life Technologies) supplemented with 10% (vol/vol) heat-inactivated fetal bovine serum (FBS; Sigma) at 37°C in an environment with 5% CO2 and 95% humidified air. Infections were performed using density gradient-purified EBs (Scidmore, 2005). Chlamydiae were cultivated in the presence of 600 ng/ml penicillin G (PenG; Sigma), 500 µg/ml spectinomycin (Spec; AlfaAesar), 1 µg/ml cycloheximide (Sigma), and 50 ng/ml anhydrotetracycline (aTc) where appropriate.

### Strain construction

Ectopic expression of TmeB was accomplished by transformation of WT or Δ*tmeB* chlamydiae with pCOMP-TmeB. pCOMP-TmeB was generated by mobilization of *tmeB* with its endogenous promoter into pComA II (Mueller et al., 2016). Amplification was accomplished using Δ*tmeA* genomic DNA and primers 694pro@NmPgfpF (5’-CGGTTCCTGGCCTTTTGCTGG GTACGGAAATACTATCTCCAGCTCAAAGC-3’) and 695@gfpNmPR (5’- GCCCCGCCC TGCCACTCATCGTTAGATATTCCCAACCGAAGAAGGATCTTCC-3’). For Δ*tmeAB*, pSUmC expressing *gfp* and *aadA* flanked by 2.2 kb of chlamydial flanking DNA was generated. Sequences 5’ of *tmeA* were amplified from L2 genomic DNA with ctl0063-SalI-S (5’-GCAAAAGAGCTGATCCTCCGTCACTGCAGGTACCGCTCCAGCGTTGCGTATTGTTTGTGG-3’) and ctl0063-SalI-AS (5’-CGTATAATGTATGCTATACGAAGTAGG AATGCCTCCGCCGAAGCAATAACTTTTAATTCC-3’) whereas sequences downstream of *tmeB* were amplified with ctl0064-SbfI-S (5’-GTATAGCATACATTATACGAAGTTA TGCATTTTCTAATAGGGAAGAGGATAAATAGCGTG-3’) and ctl0064-SbfI-AS (5’-CTTTGATCTTTCTACGGGGTCTGACGCCCGACCATTTTACTTTGGAATAAG TGTGATATC-3’). Amplicons were sequentially cloned into pSUmC using Gibson Assembly of SalI-cut or SbfI-cut plasmid, respectively. The *tmeA* and *tmeB* double deletion strain was generated by transformation of *C. trachomatis* L2 with pSUmC-Δ*tmeAB* and allelic replacement using FRAEM as described (Mueller et al., 2017). A Δ*tmeAB-tarp* triple mutant was subsequently generated using lateral gene transfer. McCoy cells were co-infected at an MOI of 5 with a 1:1 ratio of Δ*tmeAB* and Δ*tarp*. Cultures were maintained without antibiotic selection for two 48 hr passages followed by 4 serial passages in the presence of spectinomycin and penicillin G to select for isolates carrying both the *tmeAB* and *tarp* deletions. All *Chlamydia* strains were clonally isolated as described (Mueller et al., 2017) by 2 sequential limiting dilution passages in 384 plates. Chromosomal deletions were confirmed using specific primers and qPCR as described for *tarp* (Ghosh et al., 2020), or *tmeA* and *tmeB* (Mueller et al., 2016) and whole genome sequencing (SeqCenter) was employed to verify integrity of the remaining genome.

### Immunodetection

For immunoblot analyses, proteins were concentrated from purified EBs by trichloroacetic acid precipitation. Material was separated on 4 to 15% SDS-PAGE gels (Bio-Rad) and transferred to 0.45 µm PVDF membranes (Millipore). Primary antibodies used include: MOMP (Hower et al., 2009), TmeA (Hower et al., 2009), TmeB (Mueller and Fields, 2015), Tarp (Clifton et al., 2004), Tepp (kindly provided by Dr. Raphael Valdivia, Duke University), Hsp60 (A57-B9; Santa Cruz), N-WASP (30D10; Cell Signaling), and ARPC1 (13C9; Millipore). Where appropriate, immunoblots were probed with anti-phospho-tyrosine (4G10, Millipore) to detect phosphorylated Tarp and Tepp or with anti-Flag (Sigma) to detect flag-tagged proteins. We used peroxidase-conjugated secondary antibodies (Sigma) and Amersham ECL Plus (GE Healthcare UK Limited) detection reagent to visualize proteins.

### Immunoprecipitation

Co-purification of host proteins with TmeA-FT, TmeB-FT, or Cpn0678-FT was performed essentially as described (Keb et al., 2021). Briefly, HeLa cells were transfected with ectopic expression vectors and seeded in one 6-well plate each. Additional controls included cells expressing eGFP or Cpn0677-FT. Cultures were harvested in NP40 buffer (50 mM Tris pH7.5, 150 mM NaCl, 1 mM EDTA, 0.5% NP-40) as described (Mirrashidi Kathleen et al., 2015) after 24 hrs, and incubated on ice for 1 hr. Soluble fractions were applied to equilibrated Anti-Flag M2 Affinity gel (Sigma) and incubated overnight at 4°C. The resin was washed 3x with lysis buffer and FT proteins were eluted with 3X Flag peptide in PBS. 6x Laemmli buffer was added to eluates prior to separation on SDS-PAGE gels for immunoblotting.

### Chlamydial invasion assay

HeLa229 cells were prepared in 24-well plates with 12 mm coverslips, and invasion assays were performed essentially as described (Carabeo et al., 2002). Density gradient purified EBs were used at a multiplicity of infection (MOI) of 20. Where appropriate, HeLa cells were pretreated with 200 µM CK666 (all purchased from Sigma-Aldrich) for 15 min prior to infection and maintained with 200 µM CK666 during infection and subsequent incubation. All infections were carried out on ice with rocking for 1 hr then shifted to 37°C for 45 min. Cultures were then treated with 4% paraformaldehyde, and extracellular or intracellular EBs were differentially labeled with murine LPS-specific or rabbit MOMP (Hower et al., 2009) antibodies, respectively. Secondary antibody conjugated to Alexa-594 (anti-mouse) or Alexa-488 (anti-rabbit) were used for detection. Internal and external chlamydiae were enumerated in 10 fields of view. Percent EB internalization was calculated via the formula [(total EBs –external EBs)/total red EBs] x 100 = percent (%) invasion.

### Pyrene assay

Pyrene actin polymerization assays were performed as previously described (Jiwani et al., 2013). Full-length TmeB was expressed as a GST fusion in pGEX-6p-1, purified to homogeneity over GST affinity column, and eluted by cleavage of the GST-tag with PreScission protease (Sigma). Remaining components were from the commercially available pyrene actin polymerization kit (Cytoskeleton). For polymerization assays, monomeric pyrene-labeled actin was prepared by diluting lyophilized pyrene actin (Cytoskeleton) in 5mM Tris (pH 8.0) 0.2mM CaCl_2_ 0.2mM ATP (G buffer) and collection of the supernatant of a 90 minute, 100,000 xg, 4°C spin in a Beckman Optima MAX TL Ultracentrifuge using a TLA 55 rotor (Beckman Coulter). Approximately 30 µg of pyrene-labeled actin was mixed with 1-2µg of indicated proteins (TmeB, VCA, Arp2/3, GST) in a volume of 500 µl for 5 min before the addition of 1/20^th^ volume of polymerization buffer (500mM KCl, 20mM MgCl_2_, 10mM ATP). The reaction (contained in a semi-microcuvette and holder assembly) was monitored for 30 min with a LS 55 Luminescence spectrophotometer equipped with the biokinetic accessory and directed by FL Winlab software version 4.0 (Perkin-Elmer, Beaconsfield, Bucks, United Kingdom) with 2.5 nm bandwidth at 365 nm excitation wavelength and 2.5 nm bandwidth at 407 nm emission wavelength.

### Statistical analysis

Unless otherwise noted, presented data are representative of triplicate experiments where quantitative data were generated from experiments containing triplicate biological replicates. Calculation of standard deviation of the mean and assessment via Student’s t-test with Welch’s correction. Statistical analyses were performed using GraphPad Prism 6 version 6.04 (Graphpad Software Inc).

## Results

### TmeB abundance correlates with changes in invasion efficiency

**Figure 1:**
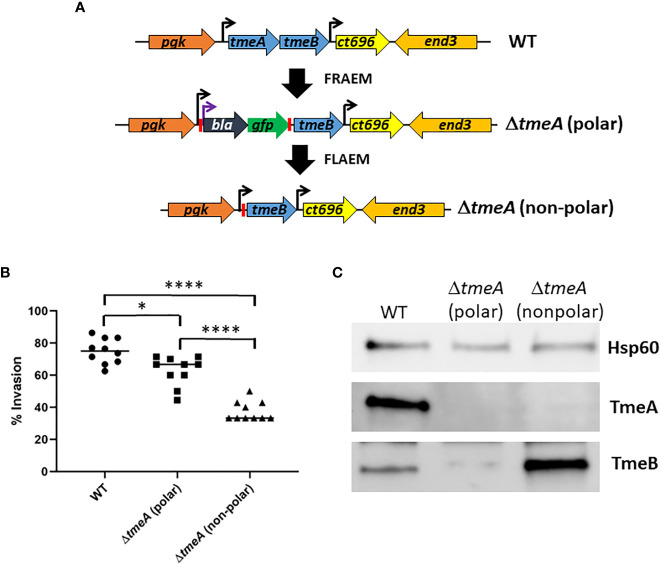
Abundance of TmeB correlates with severity of *C. trachomatis* invasion defect. **(A)** Schematic of *C. trachomatis* L2 *tmeAB* locus in WT, polar Δ*tmeA* and non-polar Δ*tmeA* strains. Endogenous (black) or engineered (purple) promoters are indicated by arrows. Engineered *loxP* sites are indicated by red bars. **(B)** HeLa monolayers were infected for 1 hr at 4°C with WT or mutant strains at an MOI of 10. Cultures were shifted to 37°C for 30 min and then paraformaldehyde fixed and processed for inside-out staining to assess invasion efficiency. Data from 10 fields of view are represented with means for percentage of internalized chlamydiae. Statistical significance was computed using a Student’s T-test with Welch’s correction (*P<0.005 and ****P<0.0001). **(C)** Immunoblot of material concentrated from equal EBs from WT, polar Δ*tmeA* and non-polar Δ*tmeA* strains. TmeA and TmeB were detected by chemilumenescence using specific antibodies and blots were probed with Hsp60-specific antibodies as a loading control.

We developed floxed allelic exchange mutagenesis (FLAEM) to enable marker-less gene deletion in *C. trachomatis*. This approach (**Figure 1A**) was previously used (Keb and Fields, 2020) to excise a *blaM*-*gfp* cassette replacing the *tmeA* locus in our original Δ*tmeA* null (Mueller et al., 2016) created by fluorescence-reported allelic exchange mutagenesis (FRAEM). The marker-induced polar effect on downstream *tmeB* was relieved, resulting in increased TmeB abundance (Keb and Fields, 2020). During the course of our TmeA functional studies, we noticed the new non-polar Δ*tmeA* (referred to previously as Δ*tmeA-lx*) strain appeared to exhibit a more robust invasion defect than the parent polar strain. We directly tested for a potential difference by performing side-by-side invasion studies (**Figure 1B**). Compared to WT, polar Δ*tmeA* reduced invasion efficiency by ca 16% whereas the non-polar strain was reduced by ca 50%. Corresponding protein abundance was examined in material concentrated from gradient-purified EBs (**Figure 1C**). As expected, immunoblot data confirmed loss of TmeA and lower abundance of TmeB in the polar Δ*tmeA* strain. Interestingly, a pronounced overabundance of TmeB was detected in non-polar Δ*tmeA* compared to WT EBs. TmeB overabundance was specific to the non-polar Δ*tmeA* strain since WT levels of TmeB were detected in strains lacking *tepp* or *tarp* (**Figure S1**). These data indicate an inverse correlation between invasion efficiency and TmeB abundance when TmeA is absent.

**Figure 2:**
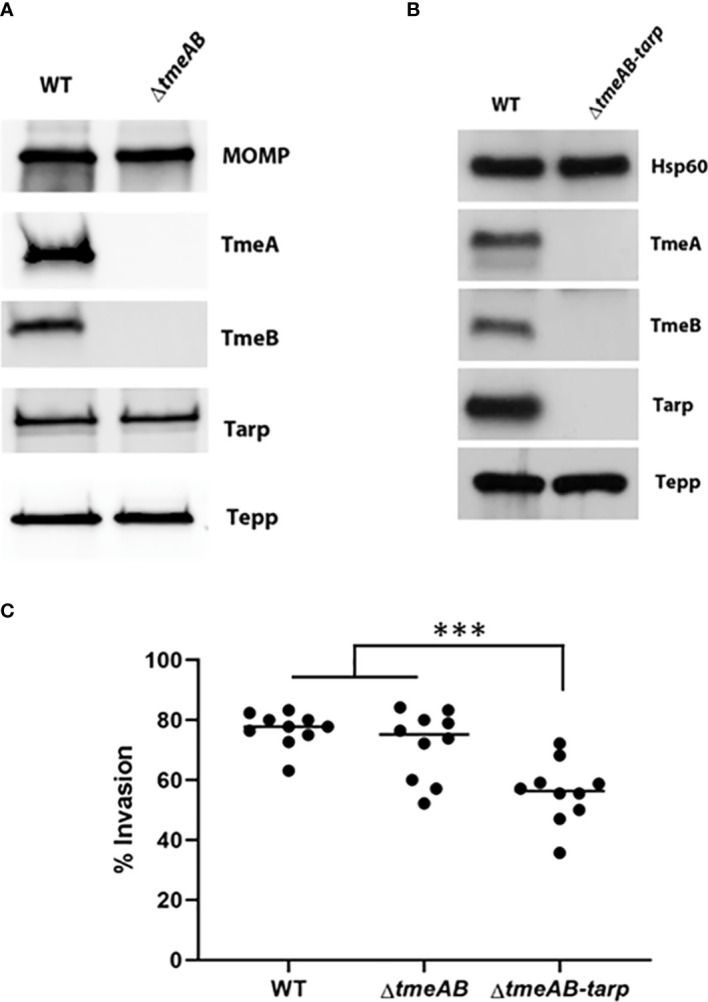
Invasion efficiencies of double and triple mutants. **(A, B)** Immunoblot analysis of material concentrated from purified WT, double mutant (Δ*tmeAB*), and triple mutant (Δ*tmeAB-tarp*) EBs. TmeA, TmeB, Tarp and Tepp were detected with specific antibodies. Antigen-specific antibodies to either MOMP or Hsp60 were used as loading controls. All proteins were visualized by chemilumenescence. **(C)** HeLa monolayers were infected for 1 hr at 4°C with WT or mutant strains at an MOI of 10. Cultures were paraformaldehyde fixed and processed for inside-out staining after 30 min at 37°C. Bacteria were enumerated over 10 fields of view and invasion efficiencies represented with means for percentage of internalized chlamydiae. Statistical significance was computed using One-way Anova (***P<0.0001).

We next generated a strain containing a complete deletion of both *tmeA* and *tmeB* to address whether the invasion defect manifested by Δ*tmeA* strains was due solely to the absence of TmeA. *C. trachomatis* L2 was transformed with pSUmC-Δ*tmeAB*, and FRAEM was employed to replace the entire coding region for *tmeA* and *tmeB* with a *gfp*-*aadA* cassette. Gene deletion was first confirmed in a clonal isolate via qPCR (**Figure S2A**) and then via immunoblot using specific antibodies to probe EB lysates (**Figure 2A**). Neither TmeA nor TmeB were detected whereas co-secreted effectors Tarp and Tepp remained unchanged compared to WT. Tarp is required for efficient invasion, and a triple mutant was generated using lateral gene transfer. Co-infections containing Spec^r^ Δ*tmeAB* and PenG^r^ Δ*tarp* (Ghosh et al., 2020) were cultivated in the presence of both antibiotics to select a recombinant strain carrying both mutations. Respective deletions of a clonal isolate were confirmed via qPCR (**Figure S2B**). Subsequent immunoblot (**Figure 2B**) analysis confirmed the loss of TmeA, TmeB, and Tarp in a single strain. Invasion efficiency of each strain was compared to WT (**Figure 2C**). While levels of Δ*tmeAB* invasion did not differ from WT, the additional loss of Tarp manifested a significant decrease in internalization efficiency. These data raise the possibility that TmeA, but not Tarp, is required for efficient invasion only in the presence of TmeB.

**Figure 3:**
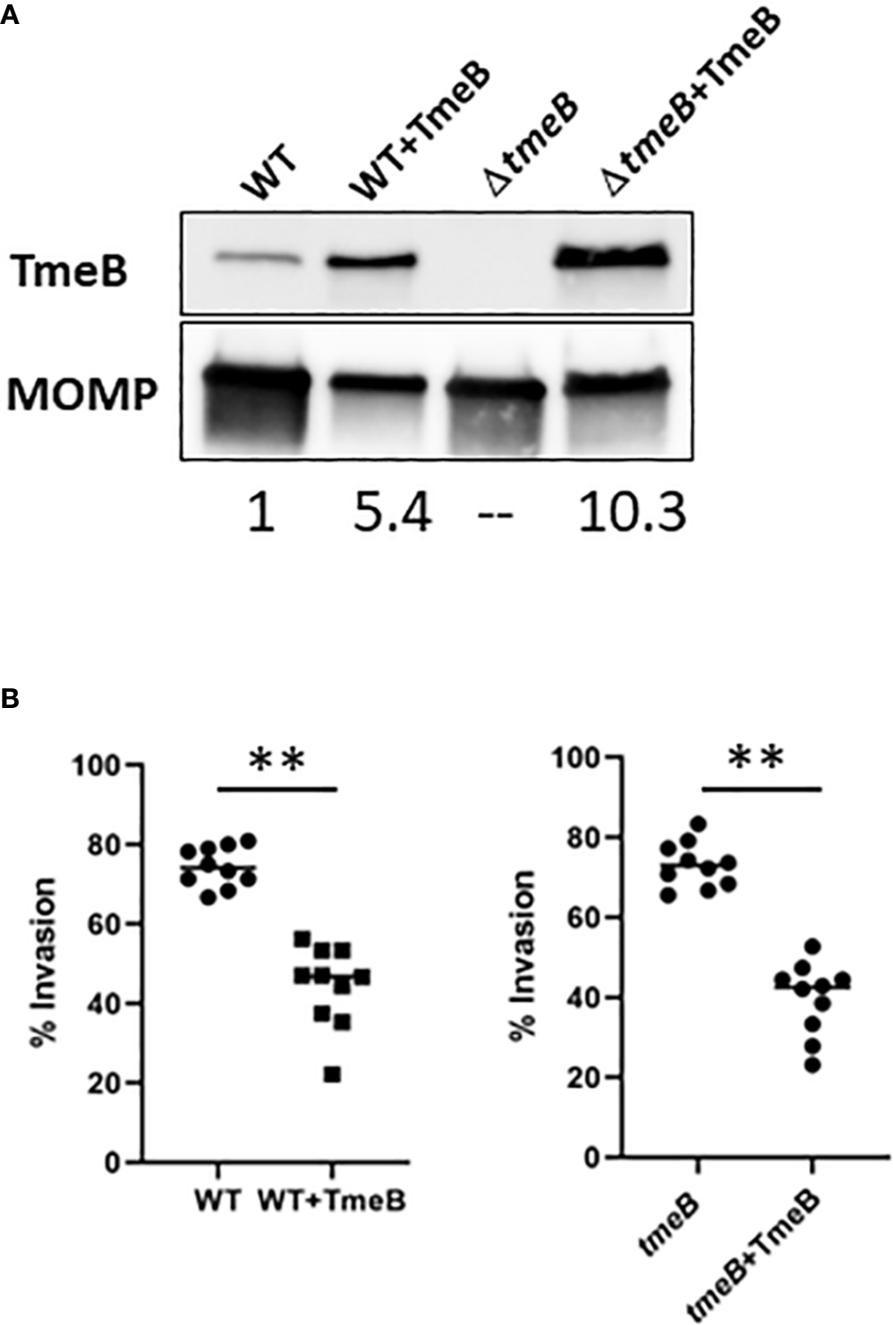
The impact of TmeB overexpression during invasion. **(A)** Immunoblot analysis of material concentrated from purified EBs from WT and Δ*tmeB* alone or expressing pCOMP-TmeB (+TmeB). Blots were probed with TmeB-specific antibodies or MOMP antibodies as a loading control and visualized via chemilumenescence. TmeB band intensities were determined using ImageJ and normalized based on MOMP signal with WT being nominally set to 1. **(B)** HeLa monolayers were infected for 1 hr at 4°C with respective strains at an MOI of 10. Cultures were paraformaldehyde fixed and processed for inside-out staining after 30 min at 37°C. Bacteria were enumerated over 10 fields of view and invasion efficiencies represented with means for percentage of internalized chlamydiae. Statistical significance was computed using a Student’s T-test with Welch’s correction (**P<0.001).

Non-physiological levels of TmeB in non-polar Δ*tmeA* also raised the possibility that TmeB could be directly detrimental in the absence of co-expressed TmeA. *C. trachomatis* WT and Δ*tmeB* were transformed with pCOMP constitutively expressing TmeB to address this possibility. A 5-10 fold increase in TmeB abundance was evident via immunoblot analysis of EB lysates (**Figure 3A**). These strains were then assayed for invasion efficiency after allowing 30 min for internalization (**Figure 3B**). As shown previously (Keb et al., 2021), loss of *tmeB* did not interfere with invasion efficiency compared to WT. The presence of excess TmeB in WT and Δ*tmeB*, however, did correlate with decreased invasion efficiency. These data implicate non-physiological levels of TmeB as a contributing factor to the observed impairment of chlamydial entry into epithelial cells.

### TmeB targets the Arp2/3 complex

**Figure 4:**
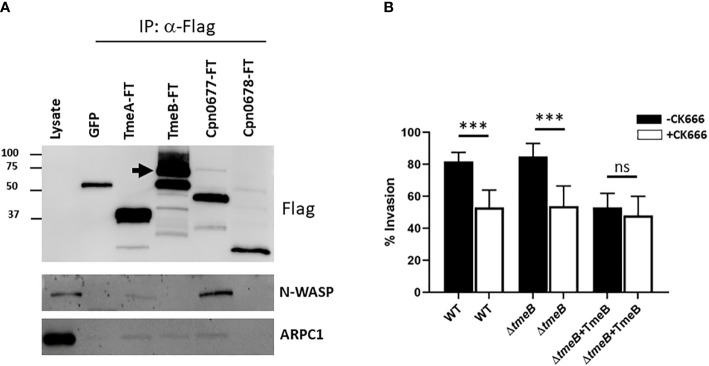
TmeB targets the Arp2/3 complex. **(A)** Flag-tagged proteins were immunoprecipitated from whole-cell lysates of HeLa cells transiently expressing GFP, TmeA-FT, TmeB-FT, Cpn0677-FT, or Cpn0678-FT for 24 hrs. Eluted material was probed in immunoblots for tagged chlamydial proteins using anti-Flag antibodies. Host proteins were detected using antigen-specific antibodies, and HeLa whole-cell lysates were included as a positive control for these antibodies. Arrow indicated position of full-length TmeB-FT. **(B)** HeLa monolayers were infected for 1 hr at 4°C with WT, Δ*tmeB* or Δ*tmeB*+TmeB strains at an MOI of 10. Infections were carried in the absence (black bars) or presence (white bars) of 200 µM CK666. Cultures were shifted to 37°C and maintained for 30 min, with or without drug, then paraformaldehyde fixed and processed for inside-out staining to assess invasion efficiency. Data are represented as mean values for percentage of internalized chlamydiae and are shown with standard deviations. Statistical significance was computed using a Student’s T test with Welch’s correction (***P<0.0001). ns, not significant.

Possibilities for TmeB-specific interference with invasion include indirect effects such as competition for the secretion chaperone Slc1 within chlamydiae or more directly by manifesting a dysregulated activity within host cells. To test the former, HeLa cultures were infected for 1 hr with WT or Δ*tmeB* strains alone or ectopically expressing TmeB. Material from whole-cell lysates was probed with phospho-tyrosine-specific antibodies to detect translocated Tarp and Tepp (**Figure S3**). Both were readily detected in all strains, indicating that negative impact of excess TmeB likely does not manifest by interfering with type III secretion. Given that operon-encoded genes are often functionally related, we next reasoned that TmeB could have an activity related to TmeA. HeLa cells were transiently transfected with plasmids encoding Flag-tagged TmeA or TmeB. HeLa cells were also nucleofected with plasmids expressing eGFP as a mock control. Plasmids encoding Flag-tagged *C. pneumoniae* Cpn0677 or Cpn0678, which are encoded by *tmeA*-syntenic genes found in *C. pneumoniae*, were used as specificity controls. Flag-tagged proteins were immunoprecipitated under mild lysis conditions using Flag-beads, and material was probed in immunoblots for N-WASP, which is a molecular target of TmeA (**Figure 4A**). We also probed for the Arp2/3 complex using antibodies specific for the ARPC1 subunit. As expected, all Flag-tagged proteins were recovered and neither N-WASP nor ARPC1 co-precipitated with Cpn0678. A cross-reactive band was detected in the GFP control with Flag antibodies, but neither N-WASP nor ARPC1 were detected. N-WASP was detected in the presence of TmeA and *C. pneumonia* Cpn0677. We also detected ARPC1 which presumably co-precipitates with the activated N-WASP complex. N-WASP did not co-precipitate with TmeB, yet ARPC1 was detected, raising the possibility that TmeB targets the Arp2/3 complex. We tested this hypothesis by assessing comparative invasion in the presence or absence of the pharmacologic Arp2/3 inhibitor CK666 (**Figure 4B**). We chose a CK666 dose sufficient to inhibit WT invasion ca. 50%. HeLa cells were infected with *C. trachomatis* WT, Δ*tmeB* or Δ*tmeB*+TmeB with or without 200 µM CK666 and processed for internalization counts at 30 min. Consistent with previous studies, cultivation with CK666 significantly decreased WT invasion. This was also detected for Δ*tmeB*. Interestingly, CK666 failed to additionally inhibit Δ*tmeB*+TmeB, indicating that the negative impact was not synergistic. Instead, the negative effect of over-expressed TmeB appears to be manifested through Arp2/3. In aggregate, these data suggest that TmeB targets the host Arp2/3 complex during invasion in an inhibitory fashion.

### TmeB interferes with Arp2/3-dependent actin polymerization

**Figure 5:**
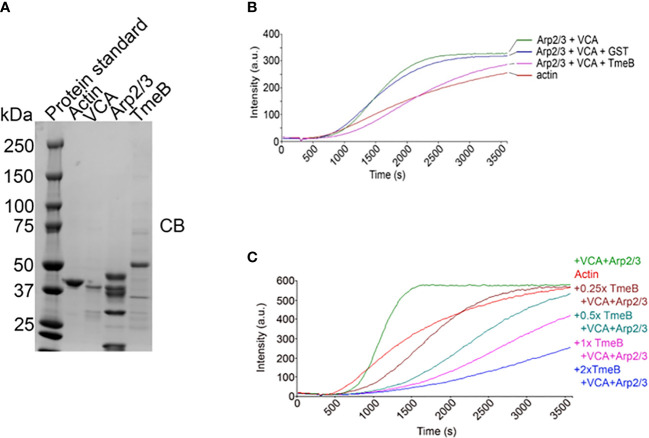
Impact of TmeB on Arp2/3-mediated actin polymerization. **(A)** Coomassie stained preparations of the purified Actin, Arp2/3 complex, the Verprolin homology, Central, Acidic domain of N-WASP, and TmeB employed in the pyrene actin polymerization assay. Increase in actin polymerization after the addition of polymerization buffer at 300 s was measured as the arbitrary fluorescence intensity (arbitrary units [a.u.] over time [s]) with excitation and emission wavelengths of 365 and 407 nm respectively. **(B)** TmeB, Arp2/3 and VCA were added individually or in combination to monomeric pyrene-labeled actin and actin alone served as a negative control. **(C)** Like the assay shown in **(A)** but with 2-fold increases in the amount of pure TmeB included in mixtures. Maximal actin polymerization occurred in the presence of Arp2/3 and VCA and this was inhibited by the presence of TmeB.

The pyrene-based actin fluorescence assay is a well-established approach to functionally investigate factors impacting the state of actin polymerization. Indeed, we have leveraged this approach to demonstrate Tarp-dependent actin polymerization (Jewett et al., 2006) and activation of N-WASP-dependent polymerization by TmeA (Keb et al., 2021). The verprolin homology, central, acidic (VCA) domain of N-WASP was used to activate Arp2/3 and test the impact of TmeB on actin polymerization. Purified preparations (**Figure 5A**) of protein components were incubated alone or in combination with pyrene-conjugated actin. As expected, the combination of Arp2/3 and VCA increased polymerization rates, as indicated by fluorescence intensity, compared to actin alone (**Figure 5B**). Addition of control GST did not interfere with this polymerization. In the presence of pure TmeB, the fluorescence intensity profile was similar to that of actin alone. TmeB-mediated inhibition of actin polymerization was dose-dependent as increasing levels of TmeB resulted in progressively dampened actin polymerization rates (**Figure 5C**). This decrease in actin polymerization depended on Arp2/3 since, in the absence of Arp2/3 and VCA, TmeB alone had no impact on polymerization rates (**Figure S4**). In aggregate, these data indicate that one function of TmeB is to antagonize branched actin polymerization by interfering with Arp2/3 activity.

**Figure 6:**
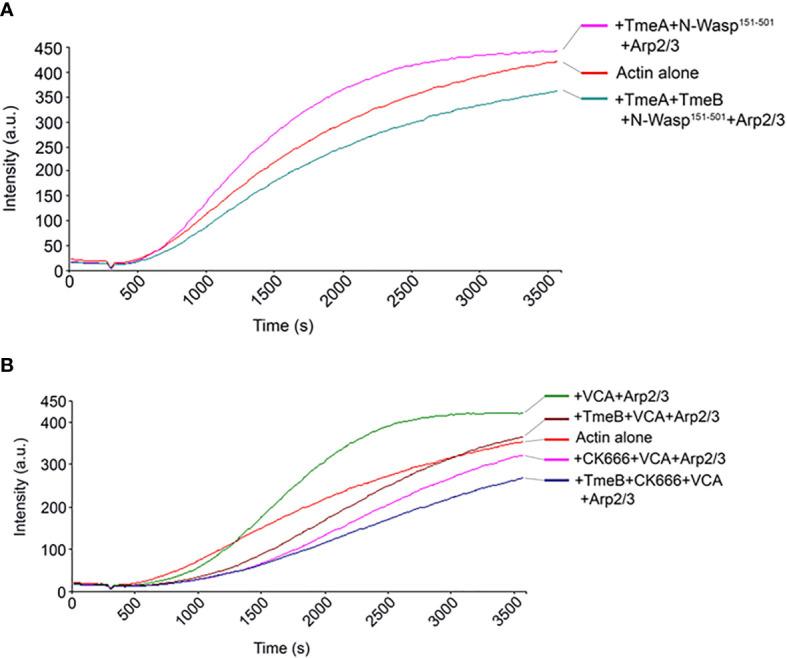
TmeB inhibition in the presence of TmeA-N-WASP or CK666. Actin polymerization was tested measured as the arbitrary fluorescence intensity (arbitrary units [a.u.] over time [s]) with excitation and emission wavelengths of 365 and 407 nm respectively. Polymerization buffer was added at 300 s. Monomeric pyrene-labeled actin and actin alone served as a negative control. **(A)** TmeA and N-WASP^151-501^ were added individually or in combination with TmeB. **(B)** TmeB, Arp2/3 and VCA were added individually or in combination with 17 µM of the Arp2/3 inhibitor CK666.

The VCA domain of N-WASP exhibits constitutive activity, and we previously used a version of N-WASP (residues 151-501) to show that TmeA activates N-WASP to promote Arp2/3-dependent actin polymerization (Keb et al., 2021). We therefore examined whether TmeB could interfere with actin polymerization stimulated by the TmeA-N-WASP complex by combining these proteins in the pyrene assay (**Figure 6A**). As expected, addition of TmeA-N-WASP with Arp2/3 enhanced fluorescence kinetics and intensity. Inclusion of TmeB interfered with this activation and slowed the overall polymerization rate. We next tested whether TmeB acts synergistically with CK666 (**Figure 6B**). Addition of low levels (17 µM) of CK666 to VCA + Arp2/3 resulted in inhibition of actin polymerization as expected. Inclusion of TmeB shifted the fluorescence profile further downward indicating a synergistic effect between CK666 and TmeB. This additive effect was not detected at a saturating concentration (100 µM) of CK666 (**Figure S4B**). Collectively, these data suggest that CK666 and TmeB-mediated inhibition of Arp2/3 may manifest via a similar mechanism.

## Discussion

TmeA and TmeB are two *C. trachomatis* early effectors that are encoded in a bi-cistronic operon, share the secretion chaperone Slc1, and are translocated into human cells during invasion via T3S (Hower et al., 2009). Both effectors also peripherally associate with host membranes through N-terminally localized helices (Bullock et al., 2012; Mueller and Fields, 2015). While we previously demonstrated that invasion efficiency of a polar Δ*tmeA* mutant differs moderately from WT (McKuen et al., 2017), invasion efficiency by the non-polar Δ*tmeA* mutant is more robustly reduced (Keb et al., 2021). We provide evidence herein that this difference in invasion efficiency is likely manifested by strain-specific differences in TmeB abundance. The elevated level of TmeB in the non-polar Δ*tmeA* strain could be due to increased message levels (Keb et al., 2018) or translation efficiency. During EB entry of a host cell, TmeA is required to recruit host N-WASP to sites of invasion (Faris et al., 2020; Keb et al., 2021). TmeA directly induces activation of N-WASP to promote Arp2/3-directed actin polymerization (Keb et al., 2021) and this activity is postulated to contribute to remodeling of the actin cytoskeleton necessary to drive chlamydial entry (Faris et al., 2020; Keb et al., 2021). Loss of TmeB does not manifest in an entry defect (Keb et al., 2021), and functional characterization of this effector has been lacking. Using genetic and biochemical approaches, we have now demonstrated that TmeB exerts an inhibitory role in Arp2/3-mediated actin polymerization. Taken together, these data suggest a regulatory axis between TmeA and TmeB, wherein TmeB is capable of negatively regulating the ability of TmeA to incite Arp2/3-dependent actin polymerization.

Epithelial cell ARPC1, but not N-WASP, co-precipitated with ectopically expressed TmeB, raising the possibility of a direct interaction with the Arp2/3 complex. Although TmeB interfered with TmeA-N-WASP-mediated Arp2/3 activation in the *in vitro* actin polymerization assay, our additional data indicate that TmeB directly inhibits Arp2/3-mediated actin polymerization. Moreover, our efforts have failed to indicate any direct interaction of TmeA and TmeB (Fields, unpublished). CK-666 is a small molecule inhibitor of the Arp2/3 complex that binds in the pocket between Arp2 and Arp3 blocking the two subunits from forming a short-pitched dimer necessary to bind actin (Hetrick et al., 2013). Our data showed that CK-666 did not synergistically inhibit invasion alongside over expression of TmeB. These data implicate the TmeB inhibitory activity manifesting during chlamydial infection. Interestingly, TmeB’s ability to inhibit the Arp2/3 complex appears to be similar to that of the host cell regulating protein Arpin. TmeB and Arpin both share an unstructured, acidic domain implicated in the negative regulation of the Arp2/3 complex (Dang et al., 2013). The TmeB acidic domain contains 19 acidic residues in the first 80 amino acids with 10 acidic residues between amino acids 21-40 of the TmeB protein. The acidic domain of Arpin was found to associate with the Arp3 subunit leading to Arp2/3 inhibition (Fregoso et al., 2022). It is intriguing to speculate that TmeB, like Arpin binds, to and disrupts a specific subunit of the Arp2/3 complex. We observed that CK666 and TmeB inhibitory effects showed synergy and could indicate that TmeB also impacts formation of the short-pitched Arp2-Arp3 dimer. Future studies will be required to determine the specificity of the interaction between the Arp2/3 complex and TmeB, and how TmeB is able to inhibit Arp2/3-mediated actin polymerization.

*C. trachomatis* utilizes several early effectors to manipulate the host actin cytoskeleton to promote invasion of epithelial cells (Caven and Carabeo, 2019). In addition to TmeA, chlamydial Tarp can alter actin polymerization dynamics directly via interactions with monomeric or filamentous actin (Jewett et al., 2006; Jiwani et al., 2012) or indirectly by nucleating host factors culminating in Rac-1 dependent Arp2/3 activation (Caven and Carabeo, 2019). Our observation that a clean *tmeA-tmeB* double mutant does not manifest an invasion defect implies a functional link between TmeA and TmeB activities. These data indicate that TmeA is only essential for invasion in the presence of TmeB. N-WASP is likely required since trans-complementation with TmeA deficient for N-WASP binding failed to restore WT levels of entry (Faris et al., 2020). Tarp-dependent entry is seemingly not linked since the *tmeA-tmeB-tarp* triple mutant showed decreased invasion efficiency. This would be consistent with the notion that TmeA and Tarp contribute independently to invasion (Faris et al., 2020; Keb et al., 2021) and that direct actin polymerization and bundling Tarp activity is the primary requirement during chlamydial entry (Ghosh et al., 2020). Analysis of the early chlamydial effectors ability to associate with the T3S chaperone Slc1 suggests a hierarchical and temporal release of early effectors during chlamydial invasion in which Tarp and TmeA are translocated first with the subsequent recycling of chaperone Slc1 being free to translocate a second wave of effectors such as Tepp and TmeB (Chen et al., 2014). The consequence of the preferred Tarp and TmeA chaperone binding and delivery may function to fine tune actin dynamics during EB invasion by initially promoting actin filament formation followed by the immediate inhibition of Arp2/3 complex by TmeB.

Why TmeB may balance TmeA activity depends on the true role of TmeA during invasion. Faris, et al. showed that the non-polar Δ*tmeA* strain failed to manifest actin-based pedestal formation, leading them to postulate that TmeA-mediated N-WASP activation leads to protrusions associated with EBs (Faris et al., 2020). Given the newly appreciated role of excess TmeB in this strain, this effect could also be spuriously manifested by excess TmeB. N-WASP is transiently recruited to sites of invasion and associated with macropinocytosis and the Bin-Amphiphysin-Rvs (BAR) domain protein Sorting Nexin 9 (SNX9) found in F-actin based pedestals present at sites of chlamydial entry (Ford et al., 2018). While SNX9 is involved in biogenesis of filipodial structures, chlamydiae appear to associate with preformed structures instead of driving *de novo* production (Jarsch et al., 2020). Interestingly, our control protein Cpn0677 also co-immunoprecipitated with N-WASP. *C. pneumoniae* Cpn0677 is not homologous to TmeA but is encoded within a syntenic locus (Hower et al., 2009). Cpn0677 (renamed secretion effector of membrane and actin dynamics, SemD) was recently proposed as a sorting platform effector where SemD induced membrane curvature to recruit SNX9 and the subsequent interaction with N-WASP drives engulfment and scission of the nascent inclusion (Spona et al., 2023). Genetic manipulation of *C. pneumoniae* is currently lagging so the model has not been confirmed genetically. If *C. trachomatis* TmeA functions similarly, TmeB could contribute to cessation of actin polymerization around the nascent inclusion. This notion is reinforced by the fact that *C. pneumoniae* Cpn 0676 is homologous to *C. trachomatis* TmeB (Hower et al., 2009) and could function similarly. Regardless, future studies will require direct chromosomal manipulation to create in-frame TmeA domain mutants to separate TmeA and TmeB functionally.

Beyond TmeA, there are additional possibilities regarding the overarching functional consequences of Arp2/3 inhibition by TmeB. Other bacterial effectors have been shown to inhibit the Arp2/3 complex. *Legionella pneumophila* kinase LegK2 is a type IV secretion system effector protein capable of inhibiting Arp2/3 through phosphorylation of the ARPC1 and Arp3 subunits (Michard et al., 2015). TmeB has yet to be implicated as a kinase, though this is an interesting area of study that remains to be investigated. The *Legionella pneumophila* type IV secretion system phosphotyrosine-phosphatase effector protein WipA has also been shown to disrupt Arp2/3-directed actin polymerization, through a different mechanism than LegK2. WipA reduces the tyrosine phosphorylation of N-WASP and Arp3 during infection resulting in a reduction of actin polymerization (He et al., 2019). Both LegK2 and WipA hijacking of the N-Wasp/Arp2/3 pathway is speculated to promote intracellular survival and protection of the *Legionella* containing vacuoles from the endocytic pathway (Michard et al., 2015; He et al., 2019). TmeB could therefore contribute to avoidance of the nascent inclusion with fusion with vesicles of the endocytic pathway. Redundant mechanisms would be required since the Δ*tmeB* strain grows similarly to WT (McKuen et al., 2017). More broadly, dysregulated actin polymerization can be detrimental via activation of cell-autonomous immunity (Mostowy and Shenoy, 2015; Garcia et al., 2020). We postulate that at least one role of TmeB is to return host cells to a basal physiological state similar to, but mechanistically distinct from, SptP of *Salmonella* Typhimurium (Fu and Galan, 1999). Activation of the Rho-GTPases Rac1 and Cdc42 triggered by invasion-promoting effectors such as SopE can activate innate immune signaling through the NOD pattern recognition receptors (Keestra et al., 2013). SptP reverses Rho-GTPase activation (Fu and Galan, 1999) to antagonize immune detection and NF-κB (Keestra et al., 2013). In aggregate, we favor a similar working model in which the delivery of TmeB into the host cell restores cytoskeletal homeostasis by resetting structural and mechanical properties of the cytoskeleton to pre-infection levels to favor chlamydial survival, growth, and immune evasion within the nascent inclusion.

## Data availability statement

The original contributions presented in the study are included in the article/**Supplementary Material**. Further inquiries can be directed to the corresponding author.

## Author contributions

KS and GK contributed equally to this work. All authors contributed to the article and approved the submitted version.
