# Supplementary material for: Chlamydia trachomatis TmeB antagonizes actin polymerization via direct interference with Arp2/3 activity
Source: Front Cell Infect Microbiol. 2023 Jul 7;13:1232391. doi: 10.3389/fcimb.2023.1232391 (PMC10360934; doi:10.3389/fcimb.2023.1232391)
Supplement: Supplementary file 1 [file DataSheet_1.pdf]

Supplemental data

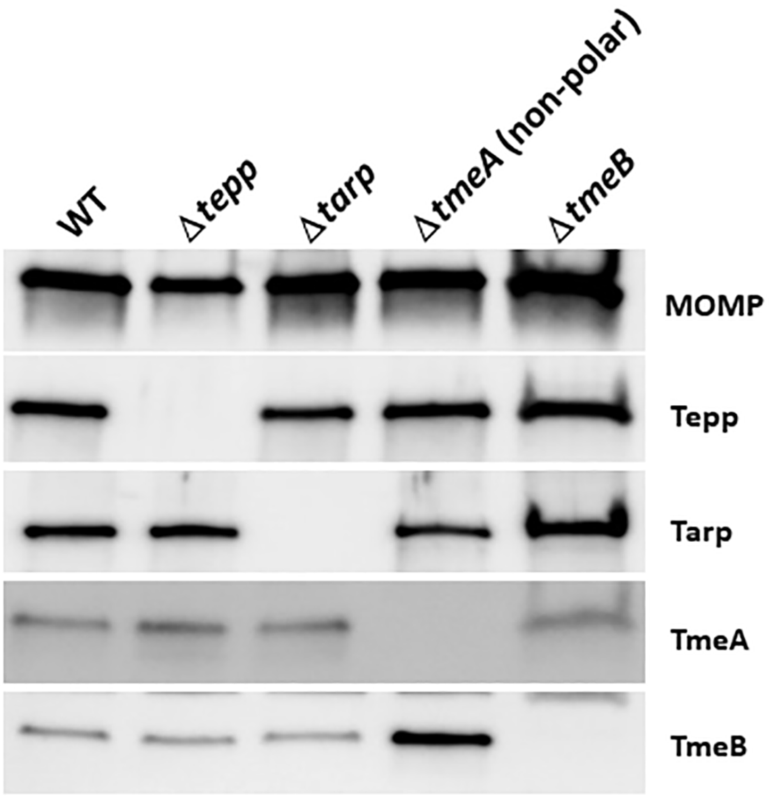

Fig S1. Proteins from equivalent numbers of DG-purified WT, Δ*tepp*, Δ*tarp*, Δ*tmeA*, Δ*tmeB* were concentrated by TCA precipitation. Material was probed with respective antigen specific antibodies and proteins visualized by chemilumenescence. MOMP was visualized as a loading control.

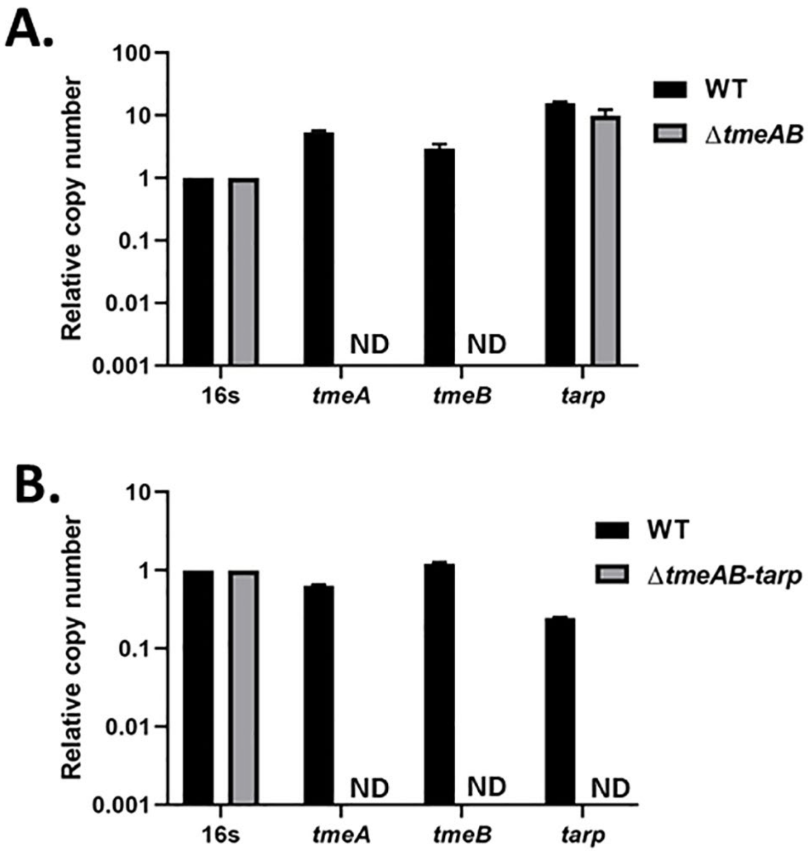

Fig. S2. Confirmation of gene deletion via qPCR. The presence or absence of *tmeA*, *tmeB*, and *tarp* was confirmed using gene-specific primers and DNA isolated from WT and the double (A) and triple (B) mutant C. trachomatis strain. Copy numbers of *tmeA*, *tmeB*, and *tarp* relative to the 16S rRNA region were determined by qPCR (data are represented as means plus SDs; n = 3). ND = none detected.

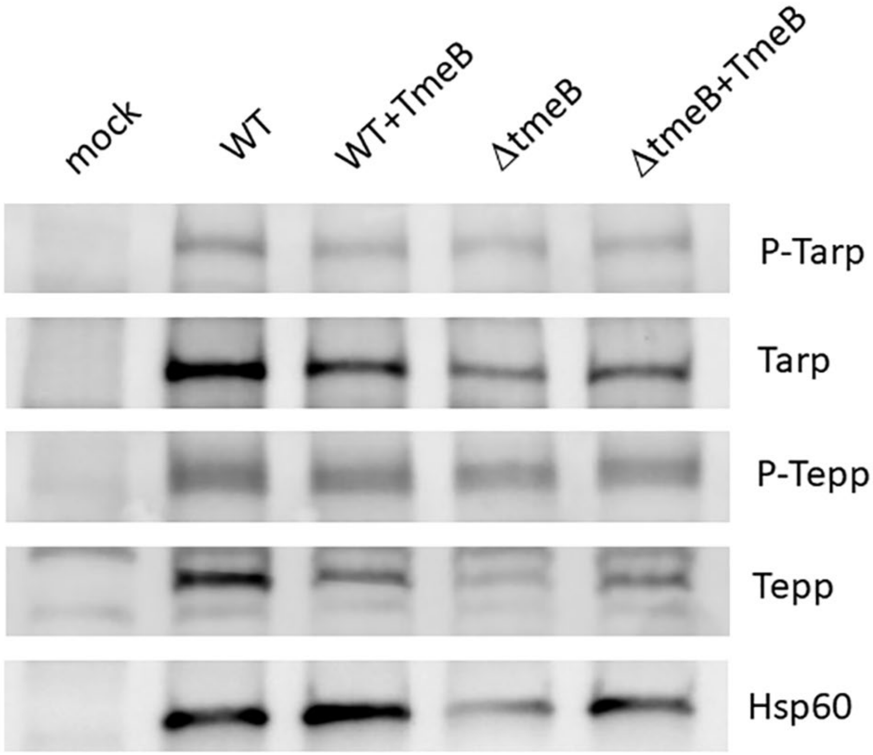

Fig. S3. Immunoblot analysis of translocated Tarp and Tepp. HeLa monolayers were mock-infected or equivalently infected with 10^7 (MOI=10) WT, Δ*tmeB*, WT+TmeB, or Δ*tmeB*+TmeB EBs. Protein concentrated from whole-culture material harvested after 1hr of infection and probed in immunoblots with phosphotyrosine-specific antibodies or with antibodies specific for Tarp or Tepp. Hsp60 was visualized as an additional loading control.

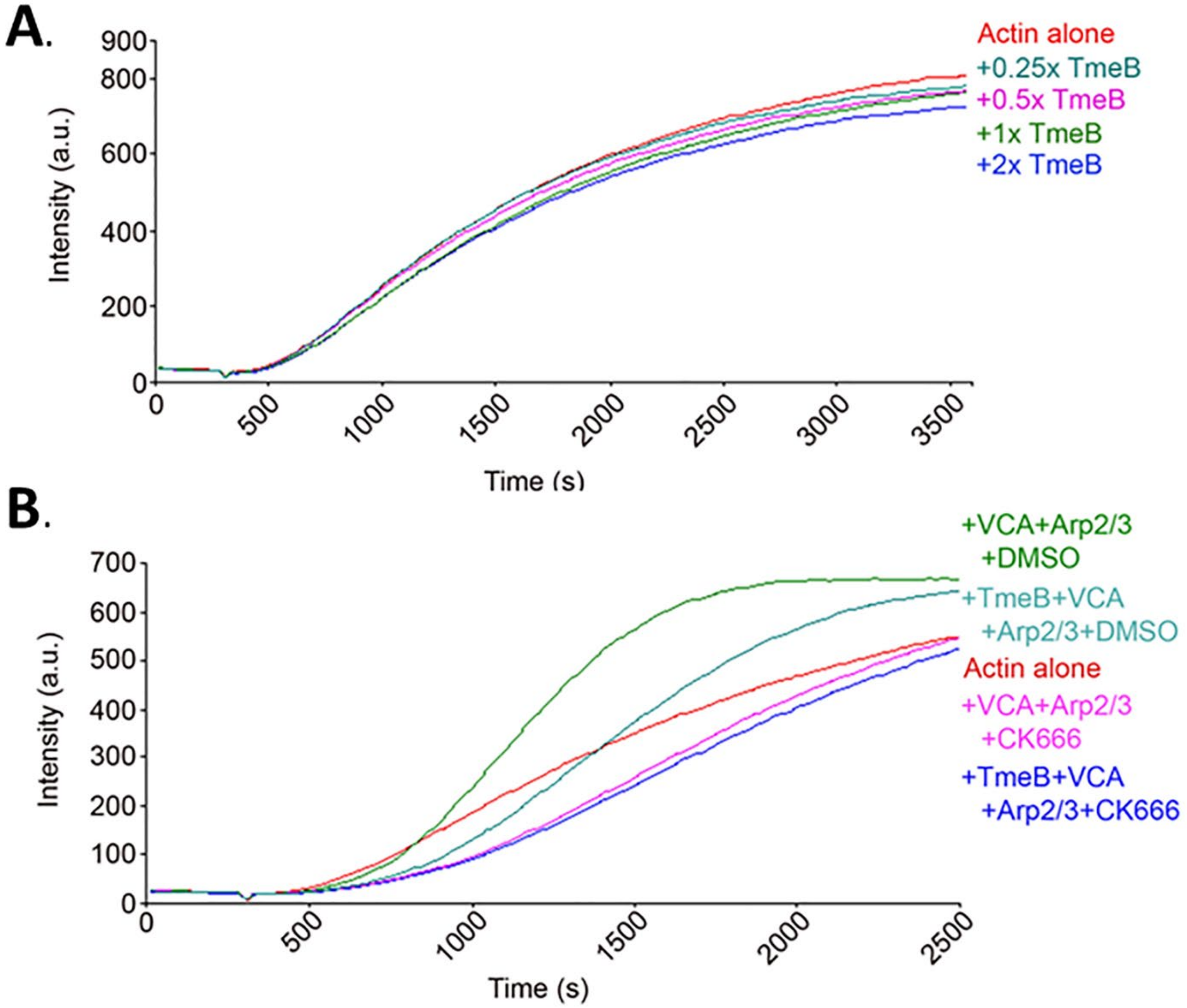

Fig S4. TmeB does not interfere with actin polymerization in the absence of Arp2/3 and does not mediated further inhibition in the presence of saturating CK666. (A) Purified actin was combined with increasing doses of purified TmeB in the pyrene actin polymerization assay. Increase in actin polymerization after the addition of polymerization buffer at 300 s was measured as the arbitrary fluorescence intensity (arbitrary units [a.u.] over time [s]) with excitation and emission wavelengths of 365 and 407 nm respectively. (B). TmeB, Arp2/3 and VCA were added individually or in combination with 100 μM of the Arp2/3 inhibitor CK666.

Actin polymerization rates and maxima were equivalent across samples.
